# Early Life Arsenic Exposure and Acute and Long-term Responses to Influenza A Infection in Mice

**DOI:** 10.1289/ehp.1306748

**Published:** 2013-08-22

**Authors:** Kathryn A. Ramsey, Rachel E. Foong, Peter D. Sly, Alexander N. Larcombe, Graeme R. Zosky

**Affiliations:** 1 Division of Clinical Sciences, Telethon Institute for Child Health Research, Subiaco, Western Australia, Australia; 2 Centre for Child Health Research, University of Western Australia, Perth, Western Australia, Australia; 3 Queensland Children’s Medical Research Institute, University of Queensland, Herston, Queensland, Australia.

## Abstract

Background: Arsenic is a significant global environmental health problem. Exposure to arsenic in early life has been shown to increase the rate of respiratory infections during infancy, reduce childhood lung function, and increase the rates of bronchiectasis in early adulthood.

Objective: We aimed to determine if early life exposure to arsenic exacerbates the response to early life influenza infection in mice.

Methods: C57BL/6 mice were exposed to arsenic *in utero* and throughout postnatal life. At 1 week of age, a subgroup of mice were infected with influenza A. We then assessed the acute and long-term effects of arsenic exposure on viral clearance, inflammation, lung structure, and lung function.

Results: Early life arsenic exposure reduced the clearance of and exacerbated the inflammatory response to influenza A, and resulted in acute and long-term changes in lung mechanics and airway structure.

Conclusions: Increased susceptibility to respiratory infections combined with exaggerated inflammatory responses throughout early life may contribute to the development of bronchiectasis in arsenic-exposed populations.

Citation: Ramsey KA, Foong RE, Sly PD, Larcombe AN, Zosky GR. 2013. Early life arsenic exposure and acute and long-term responses to influenza A infection in mice. Environ Health Perspect 121:1187–1193; http://dx.doi.org/10.1289/ehp.1306748

## Introduction

Hundreds of millions of people throughout the world are exposed to arsenic through their drinking water at doses above the World Health Organization maximum contaminant level of 10 μg/L (Mandal and Suzuki 2002). Chronic exposure to arsenic via drinking water has been shown to increase the risk of developing lung, liver, prostate, bladder, kidney, and skin cancers (Ferreccio et al. 2000; Haynes 1983; Smith et al. 1992, 1998). Arsenic exposure has also been linked to the development of nonmalignant lung diseases, including bronchiectasis, chronic bronchitis, and chronic obstructive pulmonary disease (Guha Mazumder et al. 2005; Milton et al. 2001; Smith et al. 1998). An exposure event in northern Chile, whereby the residents of Antofagasta consumed high levels of arsenic (90–860 μg/L) for two decades, revealed the significance of early life arsenic exposure in the development of respiratory disease. In the years following the peak exposure event, children from Antofagasta presented with cough, dyspnea, bronchopulmonary disease, and bronchiectasis (Borgoño and Greiber 1971; Borgoño et al. 1977; Rosenberg 1974; Zaldivar 1980). Long-term follow-up of these children revealed that the standardized mortality ratio for bronchiectasis was 46.2 [95% confidence interval (CI): 21.1, 87.7; *p* < 0.001] in those born during the peak exposure event and exposed to arsenic *in utero* and early childhood (Smith et al. 2006). Bronchiectasis is a progressive respiratory disease characterized by repeated lower respiratory tract infections and an intense inflammatory response leading to tissue damage, alterations to lung structure, and premature death in adulthood (Barker 2002). Understanding whether arsenic exposure is able to increase the susceptibility to or exacerbate responses to respiratory infections in early life may help to explain the link between arsenic exposure and the development of bronchiectasis.

There is evidence that prenatal arsenic exposure can increase the susceptibility to respiratory infections in early life. Infants in Bangladesh who had been exposed to arsenic at concentrations > 250 μg/L *in utero* had a significantly increased risk of developing lower respiratory tract infections (adjusted relative risk = 1.69; 95% CI: 1.36, 2.09) compared with those exposed to arsenic at concentrations < 39 μg/L (Rahman et al. 2011). This increase may be linked to the known immunosuppressant activity of arsenic and/or the known effects of arsenic on impairing growth and development. Exposure during pregnancy can increase inflammatory cytokines and reduce T-cell numbers in the placenta (Ahmed et al. 2011) and impair thymic development in infants (Raqib et al. 2009). *In utero* arsenic exposure is also associated with infants being born small for gestational age (Huyck et al. 2007; Rahman et al. 2009). Low birth weight is an important risk factor for the development of respiratory infections and worse lung function in childhood (Barker et al. 1991; Chan et al. 1989; Rona et al. 1993; Shaheen and Barker 1994; Vik et al. 1996) and mortality from chronic respiratory diseases in early adulthood (Stein et al. 1997; Stick 2000). We have previously shown that *in utero* exposure of mice to arsenic impairs lung growth and development, induces mucous cell metaplasia in the airways, and alters the expression of genes that regulate lung morphogenesis and mucociliary clearance in the lung, all of which could contribute to the increased risk of respiratory infections in early life (Ramsey et al. 2013a). A combination of arsenic-induced impairments to lung growth and immune development are likely mechanisms for the increased rate of respiratory infections in early childhood.

We know little, however, about how arsenic may alter the physiological response to respiratory infections. Both environmental exposure to cigarette smoke and particulate matter not only increase the susceptibility to respiratory infections but also exaggerate the inflammatory responses to respiratory infections (Arcavi and Benowitz 2004; Ciencewicki and Jaspers 2007). A previous experimental study has shown that mice exposed to arsenic in adulthood had a diminished ability to clear an influenza A (H1N1) infection, impaired CD8^+^ T-cell responses, prolonged viral carriage, and greater mortality compared with mice given influenza alone (Kozul et al. 2009). Exposure to arsenic in early life may increase the susceptibility to and exacerbate the response to respiratory infections during a period of high susceptibility in infancy. A compromised response to influenza infection in early life may have a significant effect on infant morbidity and mortality, and play an important role in the development of bronchiectasis and other chronic respiratory diseases in arsenic-exposed areas.

In the present study we investigated how exposure to arsenic *in utero* and throughout postnatal life may alter the response to an infection with influenza during infancy. We examined how arsenic modified both the acute inflammatory response to influenza infection and the long-term implications of this response for lung structure and function.

## Materials and Methods

*Animals and exposures*. C57BL/6 mice were obtained from the Animal Resources Centre (Murdoch, Western Australia). Animals were treated humanely and with regard for alleviation of suffering. All studies were conducted according to the guidelines of the National Health and Medical Research Council Australia and approved by the institutional Animal Ethics Committee of the Telethon Institute for Child Health Research. We used a previously established model of *in utero* arsenic exposure whereby pregnant mice were given drinking water containing arsenic, in the form of sodium arsenite, at 0 (control) or 100 μg/L from gestational day (GD) 8 until birth of their offspring (approximately GD19) Ramsey et al. 2013a, 2013b). After their offspring were born, maternal exposure to either arsenic or control drinking water continued.

At 1 week of age, offspring were inoculated intranasally with 10^4.2^ plaque forming units (pfu) influenza A/Mem71 (H3N1) diluted in 10 μL of virus production serum-free medium (VP-SFM; Life Technologies, Mulgrave Victoria, Australia) or the same volume of VP-SFM containing a preparation of mock-infected cells. Thus, there were four treatment groups: control (no arsenic or influenza), arsenic (no influenza), influenza (no arsenic), and arsenic plus influenza. Offspring were weaned at 4 weeks of age and continued to receive arsenic or control drinking water according to the prior exposure protocol. We measured outcomes in the offspring at 3 and 7 days postinfection (PI) (during the acute stage of influenza infection), 21 days PI (after recovery from influenza infection), and 49 days (7 weeks) PI (adulthood) (Table 1).

**Table 1:** The number of mice tested at each time point for each treatment group.

Treatment group	No. of days PI			
	>3	>7	>21	>49
Control	12	20	21	18
Arsenic	15	20	20	20
Control plus influenza	13	17	16	19
Arsenic plus influenza	13	20	16	17
PI, post­infection. Intranasal inoculation with 10^4.2^ pfu influenza A/Mem71 (H3N1) or mock-infected cells occurred at 1 week of age.				

To investigate the effects of timing of arsenic exposure on long-term lung structure and function outcomes, we exposed a separate group of adult (8 week) female C57BL/6 mice to either arsenic (100 μg/L; *n* = 7) or control (*n* = 7) drinking water for 10 weeks, the same amount of time as the mice exposed from GD8 to 8 weeks of age.

*Inflammatory cells, viral titer, and cytokines*. Cellular inflammation was measured by total and differential cell counts in bronchoalveolar lavage fluid (BALF) collected from all offspring. Whole lungs were removed from infected offspring 3 or 7 days PI for quantification of viral titer. Inflammatory cytokines [interferon-γ (IFN-γ), interleukin (IL)-6, tumor necrosis factor-α (TNF-α), and monocyte chemoattractant protein-1 (MCP-1)] were measured in BALF supernatants using a mouse inflammation Cytometric Bead Array (BD Biosciences, San Diego, CA, USA) according to the manufacturer’s instructions. We measured total protein content in BALF using the Bradford technique and the Bio-Rad Protein Assay kit (BIO-RAD, NSW, Australia) according to manufacturer instructions. Additional details are provided in Supplemental Material (p. 2).

*Thoracic gas volume and lung mechanics*. We measured lung volume and lung mechanics in offspring at 7 days, 21 days, and 7 weeks PI, and in mice exposed to arsenic only in adulthood. For measurement of lung mechanics *in vivo*, mice were anesthetized, tracheotomized, and mechanically ventilated. We used plethysmography to measure thoracic gas volume (TGV) as described previously (Janosi et al. 2006). Lung mechanics were measured using the forced-oscillation technique as described previously by Sly et al. (2003). This technique generates measures of airway resistance (R_aw_), tissue damping (G), and elastance (H). Further details are provided in Supplemental Material (pp. 3–4).

*Responsiveness to methacholine (MCh)*. Hyperresponsiveness of the respiratory system to bronchoconstricting agents, such as MCh, can reflect the presence of pulmonary inflammation or altered lung structure such as excess mucus production or increased airway smooth muscle (Lundblad et al. 2007, 2008). We measured the responsiveness to MCh in offspring at 7 weeks PI and in mice exposed to arsenic only in adulthood. Each mouse received a saline aerosol followed by increasing doses of aerosolized MCh from 0.1 to 30 mg/​mL for 90 sec (aerosols were delivered through a nebulizer). Lung function was measured every minute for 5 min after the conclusion of each aerosol dose. Further details are provided in Supplemental Material (pp. 4–5).

*Airway remodeling*. Following euthanasia, the lungs of offspring at 7 weeks PI were fixed through intratracheal instillation of 2.5% gluteraldehyde at 10 cmH_2_0. The left lung was embedded in paraffin, and 5-μm sections were cut at proximal, middle, and distal parts of the lung for airway histology. Airway sections were stained and scored blind for airway smooth muscle and airway mucous cells. Further details are provided in Supplemental Material (p. 5).

*Statistics.* Statistical analyses were conducted using SigmaPlot software (version 12.3; SPSS Science, Chicago, IL, USA). Group means were compared using two-way analysis of variance (ANOVA), with arsenic exposure and influenza exposure as independent variables, and Holm–Sidak post hoc analysis. If the interaction term was significant (*p* < 0.05), the interaction *p*-value was reported and additional analysis was performed to determine whether the effects were superadditive. We define “superadditive” as an interaction between arsenic and influenza that produces an effect significantly greater than the sum of the individual effects. To determine whether the interaction was superadditive, we divided the effect of combined arsenic and influenza by the sum of the effects of arsenic alone and influenza alone, as described by Bates et al. (2008). A ratio significantly greater than 1 (one sample, two tailed *t*-test) indicates that the interaction is superadditive. Where necessary, data were log transformed to satisfy the assumptions of normality and homoscedasticity. A *p*-value < 0.05 was considered to be significant. Data are shown as mean ± SD.

## Results

*Maternal outcomes*. We observed no significant effects of arsenic exposure on maternal body weight (*p* = 0.51), litter size (*p* = 0.89), or gestation period (*p* = 0.11). There were no effects of arsenic exposure or influenza infection on maternal water consumption (arsenic, *p* = 0.59; influenza, *p* = 0.81) (data not shown). In offspring at day 7 PI (2 weeks of age) and day 21 PI (4 weeks of age), we observed no significant differences in any outcome between male and female offspring; therefore, data for males and females were pooled.

**Figure 1:**
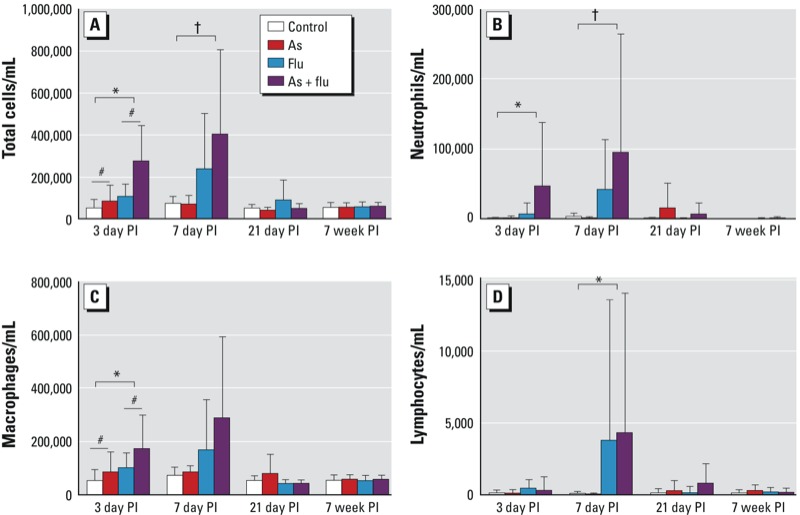
Number (mean ± SD) of total cells (*A*), neutrophils (*B*), macrophages (*C*), and lymphocytes (*D*) in BALF of mice exposed to control water, arsenic (As; 100 μg/L *in utero* and postnatal), influenza (flu), or both As and flu. Data are from day 3, day 7, and day 21, and 7 weeks post­infection (PI). The number of animals per treatment group at each time point are given in Table 1. ^#^Significant effect of As exposure (*p* < 0.05), *significant effect of flu exposure (*p* < 0.05), and ^†^significant interaction between As and flu treatments (*p* < 0.05), by two-way ANOVA with Holm–Sidak post hoc analysis.

*Inflammatory cells*. We observed a significant inflammatory response to influenza infection at days 3 and 7 PI, which was resolved at 21 days PI (Figure 1). At day 3 PI, there were effects of both arsenic and influenza on the number of total cells (arsenic, *p* = 0.002; influenza, *p* < 0.001) and the number of macrophages (arsenic *p* = 0.01; influenza *p* < 0.01) in BALF, which were additive: The mice exposed to both arsenic and influenza had a higher inflammatory response than mice treated with arsenic or influenza alone. At 3 days PI, there was a significant effect of influenza (*p* < 0.01) on neutrophil number. At day 7 PI, there was a significant interaction between arsenic and influenza on the numbers of total cells (*p* = 0.01) and neutrophils (*p* = 0.04) in BALF; that is, the number of cells present in response to influenza was significantly higher if the mice had also been exposed to arsenic. We observed a significant superadditive relationship between arsenic and influenza on the total number of cells (ratio, 2.04 ± 2.49; *p* = 0.01) and neutrophils (ratio, 2.47 ± 4.54; *p* = 0.06) in BALF at day 7 PI. We also observed a significant effect of influenza infection on lymphocyte number (*p* = 0.047) at day 7 PI. By day 21 and 7 weeks PI, the number of inflammatory cells had returned to control levels, and there was no effect of arsenic (*p* > 0.17 in all cases) or influenza (*p* > 0.15 in all cases) on the number of inflammatory cells in BALF.

**Figure 2:**
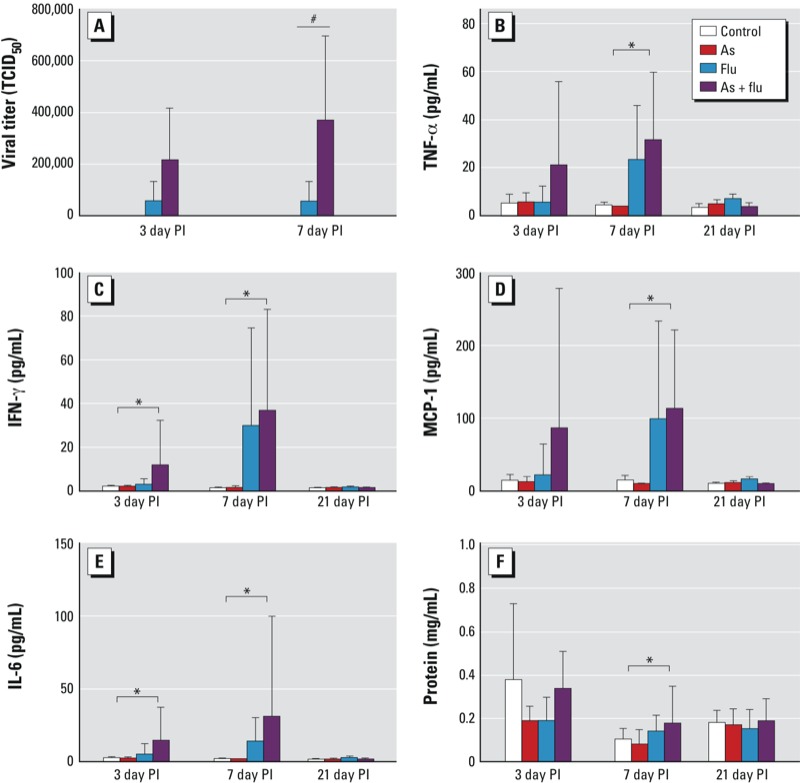
Influenza (flu) viral titer in mice exposed to flu or arsenic (As) plus flu at days 3 and 7 post­infection (PI) (*A*) and inflammatory cytokines in mice exposed to control water, As (100 μg/L *in utero* and postnatal), flu, or As plus flu at days 3, 7, and 21 PI (*B–F*). (*B*) TNF‑α, (*C*) IFN‑γ, (*D*) MCP‑1, (*E*) IL‑6, and (*F*) protein. Values are mean ± SD. The number of animals per treatment group at each time point are given in Table 1. ^#^Significant effect of As exposure (*p* < 0.05), and *significant effect of flu exposure (*p* < 0.05), by two-way ANOVA with Holm–Sidak post hoc analysis.

*Viral titer*. The median tissue culture infective dose (TCID_50_) was significantly higher in the lungs of mice exposed to both arsenic and influenza compared with those exposed to influenza alone at day 7 PI (*p* = 0.04) (Figure 2). We found some evidence of increased viral titer in the arsenic plus influenza group compared with the group that received influenza alone at day 3 PI; however, this was not statistically significant (*p* = 0.06).

*Cytokines*. We observed an increase in cytokine levels in BALF in response to influenza at day 3 and day 7 PI, which recovered to baseline levels by day 21 (Figure 2). On day 3 PI, there was an increase in IFN-γ (*p* = 0.049) and IL-6 (*p* = 0.03) in BALF from influenza-infected mice. Arsenic exposure alone had no effect on any cytokine at day 3 PI (*p* > 0.22 in all comparisons). At day 7 PI, TNF-α (*p* < 0.001), IFN-γ (*p* < 0.001), MCP-1 (*p* < 0.001), IL-6 (*p* < 0.001), and total protein level (*p* = 0.003) were increased in animals exposed to influenza, but there was no effect in animals exposed to arsenic alone (*p* > 0.087 in all comparisons). We found no significant interactions between arsenic exposure and influenza infection on cytokine levels at any time point (*p* > 0.19 in all comparisons).

*Thoracic gas volume and lung mechanics*. At day 7 PI (2 weeks of age), offspring infected with influenza had significantly lower body weight (*p* < 0.001) and higher TGV (*p* = 0.01) than uninfected offspring (see Supplemental Material, Table S1). Arsenic had no effect on body weight (*p* = 0.06) or TGV (*p* = 0.14) at this time point. To account for differences in TGV between groups, we calculated specific lung mechanics by multiplying the lung mechanics (airway resistance, tissue damping, and tissue elastance) by the TGV. At day 7 PI, arsenic exposure alone (*p* = 0.04) and influenza infection alone (*p* < 0.001) significantly increased tissue damping. There was also an additive effect of the combination of arsenic and influenza on tissue damping (see Supplemental Material, Table S1). Offspring infected with influenza had significantly higher tissue elastance (*p* = 0.002) than did uninfected offspring, but arsenic had no effect on tissue elastance (*p* = 0.17). There was no effect of arsenic (*p* = 0.48) or influenza (*p* = 0.56) on airway resistance at day 7 PI.

At day 21 PI (4 weeks of age) influenza-infected offspring were significantly smaller in weight (*p* = 0.002) than uninfected offspring (see Supplemental Material, Table S1), but we saw no effect of arsenic on body weight (*p* = 0.75). Arsenic exposure alone (*p* = 0.004) and influenza infection alone (*p* = 0.049) significantly increased TGV. In addition, we observed an additive effect of arsenic plus influenza on TGV (Supplemental Material, Table S1). To account for differences in TGV between groups, we again calculated specific lung mechanics. Both arsenic exposure (*p* = 0.009) and influenza infection (*p* = 0.038) increased tissue damping, resulting in additive effects in the group exposed to both arsenic and influenza. Offspring exposed to arsenic had significantly higher tissue elastance (*p* = 0.007) than control offspring, but influenza infection had no effect on tissue elastance (*p* = 0.20). We observed no effects of arsenic (*p* = 0.44) or influenza infection (*p* = 0.86) on airway resistance at day 21 PI.

At 7 weeks PI (8 weeks of age), males weighed significantly more than females (*p* < 0.001). Therefore, we analyzed lung mechanics separately for males and females (see Supplemental Material, Table S2). We observed additive effects of arsenic exposure (males, *p* < 0.001; females *p* = 0.04) and influenza infection (males, *p* = 0.048; females *p* = 0.04) on reducing body weight in both sexes, such that the mice exposed to both arsenic and influenza were the smallest. There were no differences in TGV between the groups in either males (arsenic, *p* = 0.09; influenza, *p* = 0.48) or females (arsenic, *p* = 0.40; influenza, *p* = 0.10). Mice exposed to arsenic had significantly higher airway resistance than mice exposed to control water (males, *p* = 0.007; females, *p* = 0.01). We saw no effect of influenza on airway resistance in either males (*p* = 0.085) or females (*p* = 0.344). Influenza-infected male offspring had significantly higher tissue damping (*p* < 0.001) and tissue elastance (*p* = 0.001) values compared with uninfected males. Also at 7 weeks PI, there was no effect of arsenic on tissue damping (*p* = 0.07) or tissue elastance (*p* = 0.086) in males, and no effect of either arsenic (tissue damping, *p* = 0.32; tissue elastance, *p* = 0.60) or influenza (tissue damping, *p* = 0.30; tissue elastance, *p* = 0.70) on tissue mechanics in female offspring.

**Figure 3:**
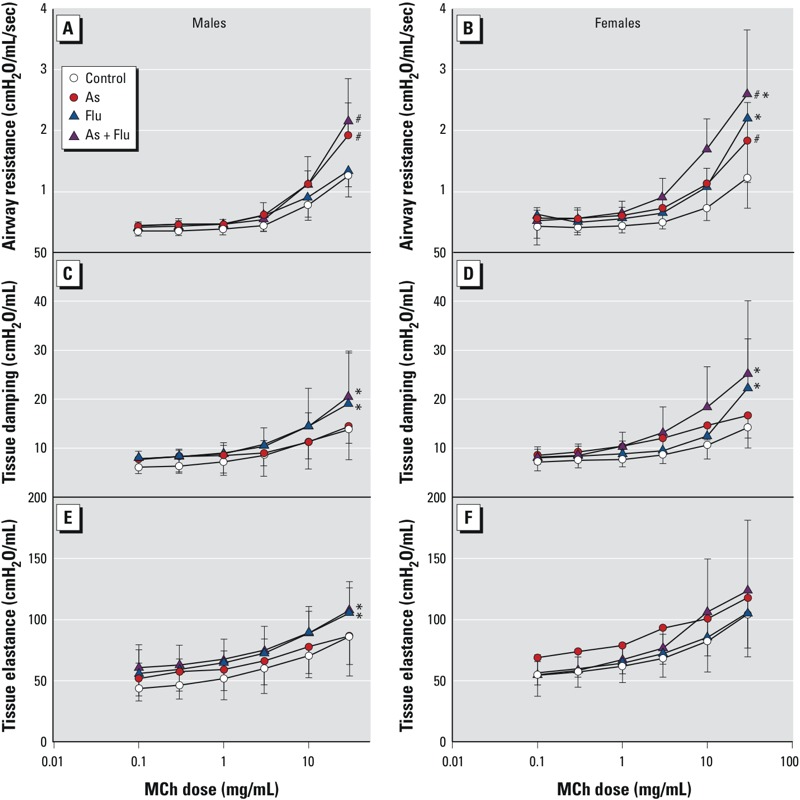
Response to methacholine (MCh) in male (*A,C,E*) and female (*B,D,F*) mice exposed to control water, arsenic (As; 100 μg/L *in utero* and postnatal), influenza (flu), or As plus flu at 7 weeks post­infection (PI) (mean ± SD). (*A,B*) Airway resistance. (*C,D*) Tissue damping. (*E,F*) Tissue elastance. The number of animals per treatment group at each time point are given in Table 1. ^#^Significant effect of As (*p* < 0.05), and *significant effect of flu (*p* < 0.05), by two-way ANOVA with Holm–Sidak post hoc analysis.

*Responsiveness to MCh.* We examined responsiveness to MCh in offspring 7 weeks PI (Figure 3). Airway resistance was increased in females exposed to arsenic (*p* = 0.04) and influenza (*p* = 0.005) at the highest dose of MCh, which was additive. In males, the maximum airway resistance to MCh was higher in arsenic-exposed offspring (*p* < 0.001) but was not influenced by influenza infection (*p* = 0.34). In both males and females, maximum tissue damping to MCh was higher in mice infected with influenza (males, *p* = 0.03; females, *p* = 0.02) compared with uninfected mice, but it was not influenced by arsenic exposure (males, *p* = 0.37; females, *p* = 0.64). In males, maximum tissue elastance to MCh was significantly higher in influenza-infected mice (*p* = 0.02) compared with uninfected controls. We observed no effect of influenza infection on maximum tissue elastance in females (*p* = 0.91) and no effect of arsenic exposure on maximum tissue elastance in males (*p* = 0.85) or females (*p* = 0.48).

**Figure 4:**
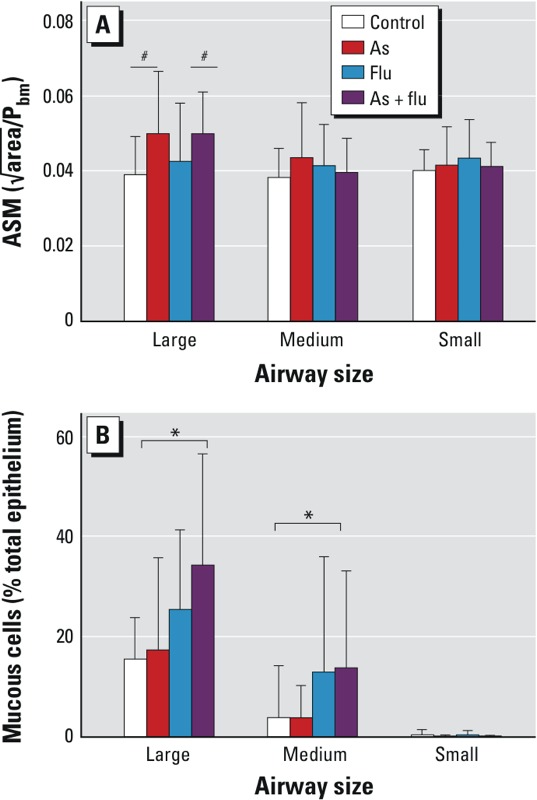
Quantification of airway smooth muscle (ASM; *A*) and airway mucus-producing (*B*) cells in large, medium, and small airways of mice exposed to control water, arsenic (As; 100 μg/L *in utero* and postnatal), influenza (flu), or As plus flu at 7 weeks post­infection (PI) (mean ± SD). To normalize for airway size, the square root of the area of the ASM layer was divided by the perimeter of the basement membrane (P_bm_). Mucous‑positive cells are presented as a percentage of the total number of epithelial cells in the airway. Airways were classified by their basement membrane perimeter (large, > 1,500 μm; medium, > 1,000 μm to < 1,500 μm; small, < 1,000 μm). The number of animals per treatment group at each time point are given in Table 1. ^#^Significant effect of As (*p* < 0.05), and *significant effect of flu (*p* < 0.05), by two-way ANOVA with Holm–Sidak post hoc analysis.

*Airway remodeling*. At 8 weeks of age, there was a greater area of airway smooth muscle in the large (*p* = 0.002)—but not medium (*p* = 0.62) or small (*p* = 0.88)—airways of arsenic-exposed mice compared with control mice (Figure 4); however, we saw no effect of influenza infection on airway smooth muscle area (*p* > 0.11). Eight-week-old mice exposed to influenza in early life had a greater number of mucus-positive cells in the large (*p* = 0.02) and medium (*p* = 0.04), but not the small (*p* > 0.05), airways compared with uninfected mice (Figure 4). Arsenic exposure did not affect the number of mucus-producing cells in any size airway (*p* > 0.35) compared with mice that received control water.

*Effects of exposure to arsenic in adulthood only*. Adult female mice exposed to arsenic for 10 weeks during adulthood were assessed for baseline lung mechanics and responsiveness to MCh. Exposure to arsenic in adulthood had no effect on baseline airway resistance (*p* = 0.63) or parenchymal mechanics (*p* > 0.56). In response to MCh, arsenic-exposed mice were not distinguishable from controls in terms of maximum airway resistance (*p* = 0.28) or parenchymal mechanics (*p* > 0.59) (Supplemental Material, Figure S1).

## Discussion

In this study we investigated the role that arsenic plays in the acute and long-term responses to early life influenza infection. In mice exposed both *in utero* and postnatally to arsenic in drinking water prior to influenza infection, the numbers of total cells and macrophages in BALF were increased at day 3 PI. At day 7 PI, arsenic exposure increased viral titer, and there was a significant superadditive interaction between arsenic exposure and influenza infection on the number of total cells and neutrophils in BALF. Although arsenic exposure altered the inflammatory cell response to influenza in BALF, arsenic exposure had no effect on the level of cytokines measured in BALF at the time points we measured. Exposure to arsenic and infection with influenza independently impaired lung mechanics in infant mice, and those mice exposed to both arsenic and influenza had the greatest deficits in lung mechanics (tissue damping). At 8 weeks of age (7 weeks PI), arsenic-exposed mice had increased airway resistance, increased airway responsiveness to MCh, and increased airway smooth muscle mass compared with control mice. We observed increased tissue damping and tissue elastance at baseline (lung mechanics measurements taken without MCh) with the maximum dose of MCh, as well as an increased number of mucus-producing cells in the airways, of influenza-exposed mice compared with control mice. Combined exposure to arsenic and influenza resulted in additive increases in airway responsiveness in 8-week-old females. These data demonstrate how exposure to arsenic in early life can alter the response to influenza infection, resulting in both acute and long-term effects on respiratory health.

Early life exposure to arsenic increased viral titer during acute influenza infection (at day 7 PI), suggesting that arsenic may alter the innate immune response to influenza. Impaired clearance of viral and bacterial infections has been reported in zebrafish exposed to arsenic through water (Nayak et al. 2007) and in adult mice exposed to arsenic via drinking water (Kozul et al. 2009). Arsenic has been shown to be an immunosuppressant in humans. For example, exposure to arsenic can alter the expression of genes and cytokines involved in immune function, T-cell receptor signaling, and inflammation in human lymphocytes (Andrew et al. 2008; Wu et al. 2003) and also alter T-cell proliferation and function (Gonsebatt et al. 1994; Hernandez-Castro et al. 2009). Urinary arsenic levels in children exposed to arsenic in drinking water were associated with reduced lymphocyte proliferation and IL-2 secretion (Soto-Pena et al. 2006). Arsenic exposure during pregnancy can increase oxidative stress and inflammation in the placenta, reduce placental T cells, and alter the expression of cord blood cytokines (IL-1β, IL-8, IFN-γ, TNF-α) (Ahmed et al. 2011). Infants exposed to arsenic *in utero* have been reported to have impaired thymic development and higher levels of fever, diarrhea, and acute respiratory infections in early life (Rahman et al. 2011; Raqib et al. 2009). In experimental studies, mice exposed to arsenic had suppressed antibody formation, inhibited T-cell proliferation and macrophage activity, and altered cytokine expression (Burns et al. 1991; Corsini et al. 1999; Lantz et al. 1994; Sikorski et al. 1989; Soto-Pena and Vega 2008; Vega et al. 2001). Arsenic has also been shown to increase ubiquitinylation and degradation of cystic fibrosis transmembrane conductance regulator (CFTR) chloride channels in the gills of killifish (Shaw et al. 2007, 2010) and human airway epithelial cells (Bomberger et al. 2012). *In utero* arsenic exposure in mice can also increase the number of mucus-producing cells and the level of calcium activated chloride channel (CLCA3) protein, which is known to regulate mucus production and secretion (Ramsey et al. 2013a). Altered CFTR and CLCA3 expression in the airways has the potential to alter the properties of mucus and airway surface liquid in the lung, resulting in impaired mucociliary clearance of respiratory pathogens and increased risk of bacterial and viral colonization (Long et al. 2006). We have shown that arsenic exposure in early life can impair the clearance of respiratory pathogens in infancy. The first years of life represent a period of high susceptibility to pathogens (World Health Organization 2011). Recurrent respiratory infections in early life can result in morbidity and mortality from respiratory disease in early childhood and may increase the risk of developing chronic respiratory disease later in life (Barker et al. 1991). In the present study, arsenic exposure exacerbated the inflammatory response to influenza in the early stages of infection. Although cytokine expression was not altered by arsenic at the time points we measured, mice developmentally exposed to arsenic had increased numbers of macrophages (day 3 PI) and neutrophils (day 7 PI) in the lung in response to influenza infection compared with mice exposed to influenza alone. Excess neutrophilia in the lungs of mice exposed to both arsenic and influenza at 7 days PI corresponded with impaired tissue mechanics (tissue damping), indicating increased resistance or closure of the peripheral airways of the lung (Lutchen and Gillis 1997; Lutchen et al. 1996). Results of the present study indicate that developmental exposure to arsenic may exacerbate (in a superadditive manner) the inflammatory response to influenza infection in early life. However, in a study of mice exposed to arsenic in adulthood, Kozul et al. (2009) found delayed and attenuated inflammatory response to influenza infection. There are two major differences between these studies that may explain the alternate responses. First, the mice in the present study were infected with influenza at 1 week of age; therefore, the peak inflammatory period occurred when the mice were young (10–14 days) during a period of immature immune development, compared with the adult infection study in which the mice were 12 weeks of age during the acute inflammatory response to influenza (Kozul et al. 2009). We have previously shown that mice infected with influenza at 3 weeks of age have higher a viral titer, a higher level of inflammation, and worse lung function than mice infected with influenza in adulthood (Larcombe et al. 2011). Second, the mice in the present study were exposed to arsenic *in utero* and early postnatal life, which has been shown to impair immune function (Ahmed et al. 2011; Raqib et al. 2009).

In the present study, we found that exposure to arsenic and influenza in early life also resulted in long-term alterations in lung structure, lung mechanics, and responsiveness to MCh. At 7 weeks PI, influenza-only mice had increased expression of mucus-producing cells in the airways, increased resistance and stiffness of the lung parenchyma (tissue damping and elastance), and increased responsiveness to MCh compared with controls. Mice exposed to arsenic only had increased airway smooth muscle mass, increased airway resistance, and airway hyperresponsiveness. Exposure to both arsenic and influenza resulted in additive increases in airway responsiveness in female mice at 8 weeks of age. Airway hyperresponsiveness to MCh can reflect a reduced airway caliber, increased airway wall thickness, increased airway smooth muscle, and/​or excess airway mucus production (Bates et al. 2008; Cockcroft and Davis 2006). These changes in airway structure result from chronic inflammation and airway remodeling (Woolcock and Permutt 2011). Airway hyperresponsiveness is a characteristic feature of chronic obstructive lung disease and often correlates with the severity of lung disease in humans (Berend et al. 2008; Cazzola et al. 1991; Ward et al. 2002). Thus, we have identified alternate mechanisms by which arsenic exposure and influenza infection are able to independently modify airway structure, lung mechanics, and responsiveness to MCh.

Here we have shown that the lung is highly sensitive to arsenic exposure in early life: Mice exposed to arsenic *in utero* and in postnatal life had significant deficits in lung mechanics at 2, 4, and 8 weeks of age, and increased airway smooth muscle and hyperresponsive airways at 8 weeks of age. In previous studies (Ramsey et al. 2013a, 2013b), we observed that exposure to arsenic *in utero* resulted in impaired lung growth, altered lung mechanics, and lung structure early in life, which resolved with age if the exposure to arsenic ceased after birth. The present study shows that continued exposure to arsenic after birth results in long-term alterations to lung mechanics. In contrast, mice exposed to the same dose of arsenic for the same period of time beginning in adulthood had no deficits in lung mechanics or airway responsiveness. Our data support the study of Lantz et al. (2009), who reported that adult mice exposed to 100 μg/L arsenic for 3 months were not hyperresponsive to MCh, whereas mice exposed to the same dose of arsenic *in utero* and early life had hyperresponsive airways.

## Conclusions

In the present study we observed that *in utero* and postnatal exposure to arsenic can increase viral load and exacerbate the inflammatory response to influenza A infection in early life. In animals developmentally exposed to both arsenic and influenza, we saw additive deficits in lung mechanics in early life and additive effects on airway responsiveness in adulthood. This combined exposure resulted in remodeling of the airways through different mechanisms, with arsenic increasing airway smooth muscle mass and influenza increasing the number of mucus-producing cells in the airways. The lungs are particularly susceptible to arsenic in early life, as evident by the lack of abnormalities seen in mice exposed to arsenic in adulthood only. We postulate that infants exposed to arsenic in early life will have an exacerbated response to respiratory infections that may result in excess inflammation and structural damage to the lung. Recurrent and exacerbated responses to respiratory infections in early life are potential mechanisms for the increased risk of developing bronchiectasis in those exposed to arsenic in early life (Smith et al. 2006). Further research into the exacerbation of respiratory infections after arsenic exposure is needed to determine whether arsenic-exposed populations are at greater risk of morbidity and mortality from lower respiratory infections.

## Supplemental Material

(487 KB) PDF
