# Supplementary material for: Early Life Arsenic Exposure and Acute and Long-term Responses to Influenza A Infection in Mice
Source: Environ Health Perspect. 2013 Aug 22;121(10):1187–93. doi: 10.1289/ehp.1306748 (PMC3801203; doi:10.1289/ehp.1306748)
Supplement: (487 KB) PDF [file ehp.1306748.s001.pdf]

Supplemental Material

Early Life Arsenic Exposure and Acute and Long-term Responses to Influenza A Infection in Mice

Kathryn A. Ramsey, Rachel E. Foong, Peter D. Sly, Alexander N. Larcombe, and Graeme R. Zosky

Table of contents

Methods……………..……………………………..……………………………..…p2

Supplemental Material, Table S1……..……………………………………………....p6

Supplemental Material, Table S2…..…………………………………………….…...p7

Supplemental Material, Figure S1……………………….………………………p8

References…………………..…………………………..…………………….….……p9

Methods

Inflammatory cells

Cellular inflammation was measured via total and differential cell counts in bronchoalveolar lavage fluid (BALf) taken from all offspring. BALf was sampled from all mice by gently washing 0.3 mL (0.5 mL in adults) of 0.9 % saline (Pfizer Pty Ltd., WA, Australia) in and out of the lungs three times and collecting the fluid and cells from the final wash. The samples were centrifuged at 400 g for 4 minutes using an Eppendorf AG centrifuge 5415D (Eppendorf, Hamburg, Germany) and the supernatant stored at -80 °C. The remaining pellet was resuspended in phosphate buffered saline (PBS) and total cell counts were performed on 10 µL of solution stained with Trypan Blue (Sigma, Australia) using a haemocytometer. The remaining suspension was spun onto slides in a Sigma 3-15 centrifuge (SIGMA©, Osterode am Harz, Germany) and stained using Leishman's stain (BDH Laboratory Supplies, Poole, England). Differential cell counts were carried out on 300 cells using light microscopy.

Viral titer

Whole lungs were removed from infected offspring 3 and 7 days post-infection for quantification of viral titer. Lungs were weighed and homogenized in VP-SFM. Lung viral titer was quantified in clarified homogenate by plaque assay. Madin-Darby canine kidney (MDCK) cells were seeded into 6-well plates. Once cells were confluent, serial 10-fold dilutions of clarified homogenate were inoculated into each well. After 2 days, wells were fixed with 5 % formaldehyde in saline and stained with 0.5 % crystal violet in methanol. We quantified influenza virus titers in whole-lung homogenate using $TCID_{50}$ (50 % tissue culture infective dose) determination.

Cytokines

Inflammatory cytokines were measured in BALf supernatants using a mouse inflammation Cytometric Bead Array (BD Biosciences, San Diego, CA, USA) as per the manufacturer's instructions. Measurement of total protein content of the BALf was carried out using the Bradford technique employing the Bio-rad Protein Assay kit according to manufacturer instructions (BIO-RAD, NSW, Australia).

Thoracic gas volume and lung mechanics

Lung volume and lung mechanics were measured in offspring at 7 days, 21 days and 7 weeks post-infection, and in mice exposed to arsenic in adulthood only. To measure lung function *in vivo*, mice were anaesthetized by intra-peritoneal injection of a mixture containing xylazine (1 mg/mL; Troy Laboratories, New South Wales, Australia) and ketamine (20 mg/mL; Troy Laboratories, New South Wales, Australia) at a dose 0.1 mL/10 g body weight. Mice were tracheotomised and a 10 mm long tracheal cannula inserted (23G stainless steel for 2 and 4 week old mice; 1.26 mm outer diameter polyethylene tube for mice 8 weeks or older) and secured with suture. Mice were ventilated (MiniVent, Harvard Apparatus, Germany) at a tidal volume of 10 mL/kg, respiratory rate of 400 breaths per minute and positive end expiratory pressure of 2 cmH_2O.

Plethysmography was used to measure thoracic gas volume (TGV) as described previously (Janosi et al. 2006). Briefly, the trachea was occluded at end expiration (transrespiratory pressure, P_{rs} = 0 cmH_2O) and the intercostal muscles were stimulated with intramuscular electrodes to induce inspiratory efforts. Six 20 V pulses of 2-3 ms in duration were delivered over a 6 s period while recording changes in tracheal pressure and plethysmograph box pressure.

TGV was calculated using Boyle's law after correcting for the impedance and thermal properties of the plethysmograph (Janosi *et al.* 2006).

Lung mechanics were measured using the forced-oscillation technique as described previously (Sly et al. 2003). The forcing function (9 frequencies from 4 – 38 Hz) was generated by a loudspeaker and delivered to the animal via a wave tube during pauses in ventilation. The respiratory system impedance spectrum (Z_{rs}) was measured and a 4-parameter model with constant phase tissue impedance was fitted to the data to partition Z_{rs} into components representing the mechanical properties of the airways and parenchyma (Hantos et al. 1992). This model allowed the calculation of airway resistance (R_{aw}) and inertance (I_{aw}) and coefficients of tissue damping (G) and elastance (H). The resistance and inertance of the tracheal cannula were subtracted from R_{aw} and I_{aw} respectively. As most of the inertance is contained in the tracheal cannula, values of I_{aw} were insignificant and not reported.

Responsiveness to Methacholine

Hyper-responsiveness of the respiratory system to bronchoconstricting agents, such as methacholine, can reflect the presence of pulmonary inflammation or altered lung structure such as excess mucous production or increased airway smooth muscle (Lundblad et al. 2007, 2008). Airway responsiveness to methacholine (MCh) was measured in offspring at 7 weeks post-infection and mice exposed to arsenic as adults only. Methacholine was prepared using Acetyl - β - methylcholine chloride (minimum 98%) (Sigma-Aldrich, St Louis, MO, USA) dissolved in sterile saline to concentrations of 0.1, 0.3, 1, 3, 10 and 30 mg/mL. Mice received a saline aerosol followed by increasing doses of aerosolized MCh from 0.1 to 30 mg/mL for 90 seconds. Lung

function was measured every minute for 5 minutes after the conclusion of each aerosol. Dose response curves were constructed from the maximal response per dose of MCh.

Airway remodeling

Following euthanasia, the lungs of offspring at 7 weeks post-infection were fixed through intratracheal instillation of 2.5 % gluteraldehyde at 10 cmH_20. The left lung was isolated and bisected into superior and inferior portions at the entrance of the left primary bronchus. The inferior portion of the left lung was embedded with the bisected face down to obtain transverse cross sections of the primary bronchus for airway morphology. Airway sections, 5µm thick, were cut at proximal, middle and distal parts of the lung for airway histology. To calculate the area of the airway smooth muscle (ASM) airway sections were stained with Masson's Trichrome (Sigma Aldrich, Castle Hill, NSW). The ASM layer was traced at 100x magnification in circular airways using stereological software (newCAST, Visiopharm, Hørsholm, Denmark) and a motorized microscopic stage. To normalize for airway size, the square root of the area of ASM layer was divided by the perimeter of the basement membrane (P_{bm}). The histological scoring was performed by the corresponding author who was blind to the treatment group of each sample until final analysis was performed. To calculate the number of mucous producing cells airway sections were stained with Alcian Blue (Sigma Aldrich, Castle Hill, NSW) to measure mucous producing cells. Mucous cell expression was calculated as the percentage of mucous positive cells divided by the total number of epithelial cells in the airway. Airways were classified by their basement membrane perimeter (large >1,500 µm, medium > 1,000 µm, small <1,000 µm) (Hirota et al. 2006).

Supplemental Material, Table S1: Body weight, thoracic gas volume (TGV) and baseline lung mechanics (R_{aw} = airway resistance, G = tissue damping, H = tissue elastance) of mice exposed to control water, arsenic (100 µg/L *in utero* and postnatal), influenza or both arsenic and influenza at day 7 and day 21 post-infection (mean ± SD).

**Treatment group**	**Body weight (g)**	**TGV (ml)**	**R_{aw} ($hPa.s^{-1}$)**	**G (hPa)**	**H (hPa)**
Day 7 post-infection control (n = 20)	6.68 ± 0.54	0.13 ± 0.01	0.090 ± 0.02	4.52 ± 0.82	17.8 ± 4.41
Day 7 post-infection arsenic (n = 20)	7.13 ± 0.58	0.14 ± 0.02	0.089 ± 0.02	5.23 ± 0.97#	20.6 ± 4.56
Day 7 post-infection influenza (n = 17)	6.28 ± 0.81*	0.15 ± 0.02*	0.094 ± 0.02	5.70 ± 1.34*	23.3 ± 6.39*
Day 7 post-infection arsenic + influenza (n = 20)	6.45 ± 0.81*	0.15 ± 0.02*	0.091 ± 0.02	5.99 ± 1.78#*	23.9 ± 7.69*
Day 21 post-infection control (n = 21)	14.77 ± 2.05	0.20 ± 0.04	0.101 ± 0.02	3.62 ± 0.79	15.1 ± 3.44
Day 21 post-infection arsenic (n = 20)	14.53 ± 2.36	0.24 ± 0.07#	0.108 ± 0.05	4.13 ± 1.26#	18.0 ± 6.07#
Day 21 post-infection influenza (n = 16)	13.20 ± 1.79*	0.22 ± 0.06*	0.104 ± 0.04	4.23 ± 1.43*	16.0 ± 4.74
Day 21 post-infection arsenic + influenza (n = 16)	13.14 ± 1.31*	0.30 ± 0.03#*	0.103 ± 0.04	5.31 ± 1.11#*	19.7 ± 4.99#

Two way ANOVA with Holm-Sidak post hoc analysis; # indicates a significant effect of arsenic; * indicates a significant effect of influenza; $p < 0.05$.

Supplemental Material, Table S2: Body weight, thoracic gas volume (TGV) and baseline lung mechanics (R_{aw} = airway resistance, G = tissue damping, H = tissue elastance) of male and female mice exposed to control water, arsenic (100 µg/L *in utero* and postnatal), influenza or both arsenic and influenza 7 weeks post-infection (mean ± SD).

**Treatment group**	**Body weight (g)**	**TGV (ml)**	**R_{aw} (hPa.s.L^{-1})**	**G (hPa.L^{-1})**	**H (hPa.L^{-1})**
Adult males control (n = 9)	24.23 ± 1.31	0.34 ± 0.03	373.1 ± 64.5	10478 ± 1059	41438 ± 7574
Adult males arsenic (n = 10)	22.08 ± 0.79#	0.33 ± 0.06	441.4 ± 66.5#	11130 ± 948	45364 ± 5286
Adult males influenza (n = 9)	23.43 ± 1.25*	0.35 ± 0.07	415.8 ± 53.4	12004 ± 1874*	49160 ± 4512*
Adult males arsenic + influenza (n = 7)	21.39 ± 0.70#*	0.30 ± 0.05	480.5 ± 82.3#	13211 ± 1799*	53085 ± 8458*
Adult females control (n = 9)	19.00 ± 0.98	0.31 ± 0.06	383.3 ± 56.6	11097 ± 1088	47576 ± 5032
Adult females arsenic (n = 10)	17.91 ± 1.29#	0.29 ± 0.05	461.9 ± 87.0#	11400 ± 602	49478 ± 5590
Adult females influenza (n = 10)	17.92 ± 0.84*	0.33 ± 0.06	435.5 ± 106.5	11679 ± 1173	47343 ± 7118
Adult females arsenic + influenza (n = 10)	17.23 ± 1.78#*	0.32 ± 0.04	500.5 ± 74.1#	12274 ± 1961	47992 ± 8886

Two way ANOVA with Holm-Sidak post hoc analysis; # indicates a significant effect of arsenic; * indicates a significant effect of influenza; $p < 0.05$.

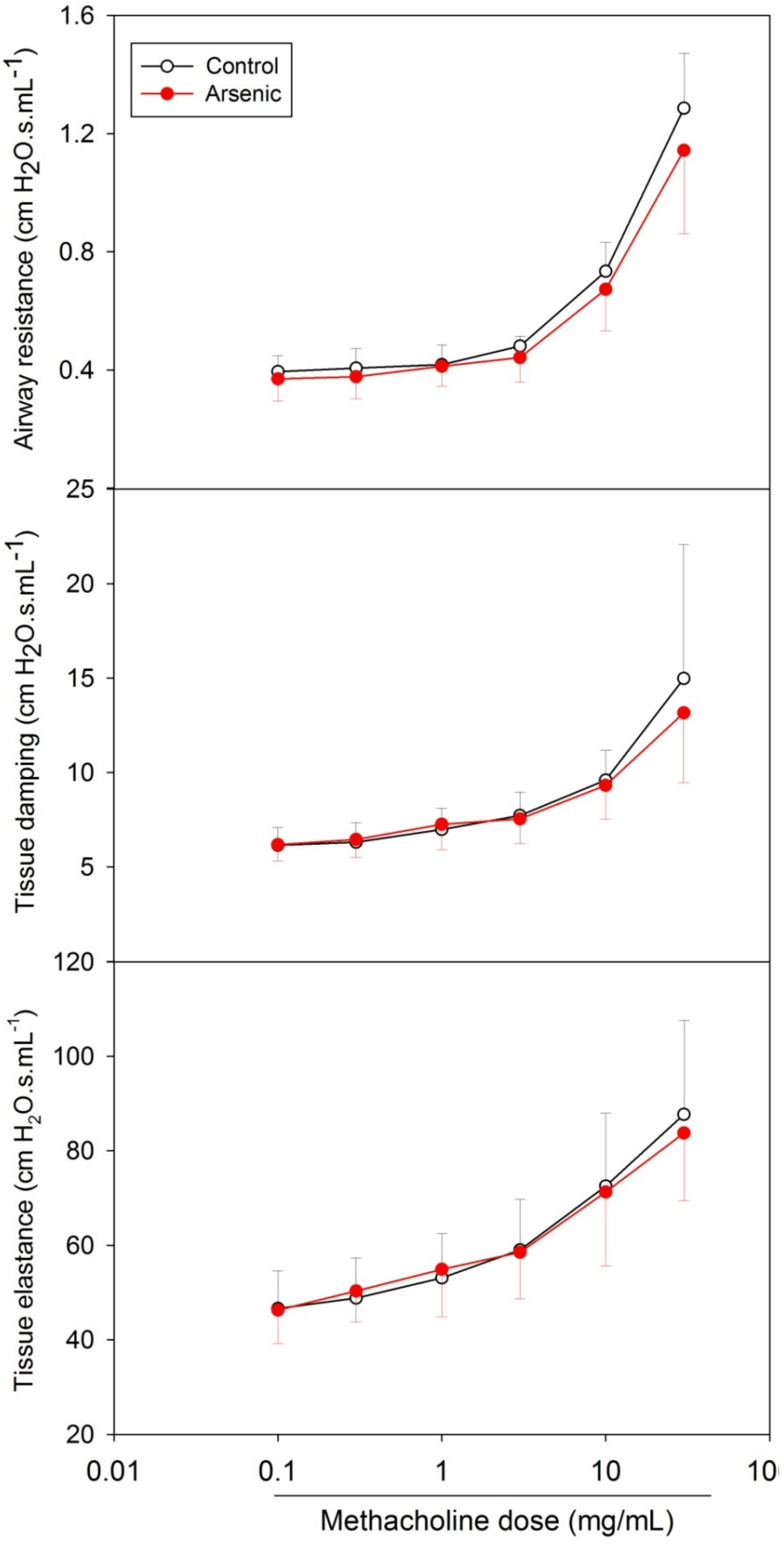

Supplemental Material, Figure S1: Response to methacholine of female mice exposed to control water or arsenic in adulthood only (100 µg/L from 8 weeks of age to 18 weeks) (mean ± SD). Two way ANOVA with Holm-Sidak post hoc analysis; bars with # indicates a significant effect of arsenic; brackets with * indicates a significant effect of influenza; $p < 0.05$.

References

Hantos Z, Daroczy B, Suki B, Nagy S, Fredberg JJ. 1992. Input impedance and peripheral inhomogeneity of dog lungs. J Appl Physiol 72(1):168-178.

Hirota JA, Ellis R, Inman MD. 2006. Regional differences in the pattern of airway remodeling following chronic allergen exposure in mice. Respir Res 7:120.

Janosi TZ, Adamicza A, Zosky GR, Asztalos T, Sly PD, Hantos Z. 2006. Plethysmographic estimation of thoracic gas volume in apneic mice. J Appl Physiol 101(2):454-459.

Lundblad LKA, Thompson-Figueroa J, Allen GB, Rinaldi L, Norton RJ, Irvin CG, et al. 2007. Airway Hyperresponsiveness in Allergically Inflamed Mice. Am J Respir Crit Care Med 175(8):768-774.

Lundblad LKA, Thompson-Figueroa J, Allen GB, Rinaldi L, Norton RJ, Irvin CG, et al. 2008. Mucous Obstruction and Airway Hyperresponsiveness in Mice. Am J Respir Crit Care Med 177(10):1171-1172.

Sly P, Collins R, Thamrin C, Turner D, Hantos Z. 2003. Volume dependence of airway and tissue impedance in mice. J Appl Physiol 94:1460-1466.
